# Left dorsomedial prefrontal cortex hyperactivation reflects early compensation for emerging social cognition difficulties in multiple sclerosis

**DOI:** 10.3389/fpsyg.2026.1905506

**Published:** 2026-09-29

**Authors:** Sara Isernia, Alice Pirastru, Diego Michael Cacciatore, Federica Rossetto, Giulia Smecca, Alessio Pedrini, Marco Rovaris, Elena Toselli, Valeria Blasi, Francesca Baglio

**Affiliations:** 1 IRCCS Fondazione Don Carlo Gnocchi ETS, Milan, Italy; 2 Department of Psychology, Università Cattolica del Sacro Cuore, Milan, Italy

**Keywords:** executive functions, multiple sclerosis, social cognition, task fMRI, theory of mind

## Abstract

**Background:**

Social cognition (SC) has emerged as a relevant neurocognitive domain in people with Multiple Sclerosis (pwMS), given its impact on everyday functioning. However, the neural mechanisms supporting SC performance in pwMS remain underexplored, highlighting the need for ecologically valid assessment paradigms capable of capturing both behavioral and neural aspects of social-cognitive functioning. In this study, we addressed this gap by investigating the clinical application of an fMRI-adapted version of the Edinburgh Social Cognition Test (ESCoT) to explore the neural correlates of SC functioning in pwMS.

**Methods:**

In total, 59 subjects (27 pwMS, mean EDSS = 3.74, and 32 healthy controls) were enrolled in this study and underwent a comprehensive neuropsychological assessment, including SC tests, and the fMRI-adapted version of the ESCoT.

**Results:**

No significant between-group differences were observed during the fMRI task performance. Across groups, the paradigm elicited activation within a network involving the bilateral middle temporal poles, left dorsomedial prefrontal cortex, and precuneus, regions consistently implicated in social-cognitive processing. Within the pwMS group, a significant association was identified between neural activation and behavioral SC performance (rho = −0.447, *p* = 0.032), linking poorer SC performance with greater activation of the left dorsomedial prefrontal cortex.

**Conclusion:**

The findings support the applicability of the ESCoT as an ecologically valid neuropsychological paradigm for capturing the neural processes underlying SC abilities in a clinical population. The observed relationship suggests that increased activation of an executive neural hub may support SC proficiency in pwMS, potentially reflecting an early compensatory strategy.

## Introduction

Social cognition (SC) impairment in people with Multiple Sclerosis (pwMS), despite its substantial impact on individuals’ everyday social functioning, remains relatively underexplored compared to other neurocognitive domains (Chalah and Ayache, 2020). Among the various facets of SC, Theory of Mind (ToM), the capacity to understand one’s own and others’ emotions and intentions, has consistently emerged as being affected in pwMS (Lin et al., 2021; Henry et al., 2022; Rossetto et al., 2025). Even so, as highlighted by recent meta-analyses, there is great variability in the prevalence and severity of ToM deficits across studies, leading to heterogeneity in SC impairment, which can be ascribed to different disease phenotypes, disability levels, cognitive reserve, and individual neuropsychological profiles (Chalah and Ayache, 2020; Lin et al., 2021; Henry et al., 2022). Furthermore, the neural mechanisms underlying these deficits remain far from understood. Although recent studies have begun to address this issue (Chalah and Ayache, 2020), the complexity of the neural networks involved in ToM and the clinical heterogeneity of MS contribute to a fragmented and nuanced picture.

Accumulating evidence has linked SC deficits in MS to structural brain alterations, including gray matter (GM) volume reduction (Chalah and Ayache, 2020; Mike et al., 2013; Batista et al., 2017; Ciampi et al., 2018; Pitteri et al., 2019; Yokote et al., 2021) and disruptions in white matter (WM) microstructural integrity (Mike et al., 2013; Batista et al., 2017; Isernia et al., 2020). Notably, the amygdala and cingulate cortex have been repeatedly implicated as key regions associated with these impairments (Batista et al., 2017; Pitteri et al., 2019; Chalah and Ayache, 2017). More recently, functional neuroimaging studies have highlighted altered neural activation patterns and disrupted connectivity in regions such as the prefrontal cortex, temporo-parietal junction, superior temporal sulcus, and amygdala (Chalah and Ayache, 2017; Bisecco et al., 2019; Golde et al., 2020; Labbe et al., 2020; Isernia et al., 2022; Koubiyr et al., 2022; Rahnemayan et al., 2025). Nevertheless, neuroimaging findings reported in the literature remain inconsistent, with studies describing both hypo- and hyperactivation of brain regions involved in SC reasoning depending on disease stage, disability level, and task characteristics, further reflecting the marked inter-individual heterogeneity of pwMS. Despite this, the application of ecologically valid paradigms capable of integrating behavioral assessment with neural measures remains limited in this population, hindering the exploration of direct associations between ToM performance and its neural correlates during task-based fMRI in pwMS.

To address this gap, the Edinburgh Social Cognition Test (ESCoT), a newly developed ecological ToM assessment (Baksh et al., 2018), has been adapted for use in fMRI to explore the ability to interpret social dynamics based on stimuli resembling the complexity of everyday social relationships (Isernia et al., 2024; Isernia et al., 2022). This task presents video clips depicting social interactions that either conform to or violate social norms, allowing for the capture of neural responses related to ToM reasoning and physical inference. The combination of ecological behavioral assessment and functional neuroimaging offers a promising approach to investigating the neural substrates of SC functioning in pwMS. In particular, the study of task-related neural activation may provide a more sensitive characterization of individual differences in SC functioning, especially in mildly disabled patients in whom subtle alterations may not yet be clinically evident.

Within this framework, the present study aims to explore the neural mechanisms associated with SC in pwMS using the ESCoT paradigm and to further investigate its validity as an ecologically valid fMRI task (Isernia et al., 2024; Isernia et al., 2022) in a clinical population. Specifically, the study focuses on identifying potential compensatory strategies directly associated with ToM ability in pwMS. Considering the marked heterogeneity of SC impairment in pwMS, we further aimed to investigate whether possible differences in neural recruitment during ToM reasoning are associated with behavioral measures of SC performance.

## Methods

This study utilized a cross-sectional design and was conducted from September 2023 to May 2025 at the IRCCS Don Gnocchi Foundation of Milan. The research adhered to the ethical principles of the revised Declaration of Helsinki. The study protocol and procedures received approval from the Local Ethics Committee (*protocol number 08_23/02/2022*).

### Participants

PwMS and sex-, age-, and education-matched healthy controls (HCs) were invited to participate in the study.

Neurologists from the Multiple Sclerosis (MS) wards of the IRCCS Fondazione Don Carlo Gnocchi clinic introduced the study to eligible outpatients during periodic medical examinations. Specific inclusion criteria were as follows: (1) diagnosis of MS based on McDonald’s criteria (Thompson et al., 2018); (2) more than 4 years of education; (3) absence of relapse in the last 3 months; (4) absence of major psychiatric comorbidity; (5) absence of severe auditory and/or visual impairment (corrected or uncorrected) that could impact the neuropsychological assessment and the fMRI task performance; and (6) absence of contraindications to MRI scanning.

HCs were contacted by consulting retrospective databases of healthy people involved in previous research studies. Eligible participants were selected based on the following criteria: (1) absence of neurological and/or major psychiatric illness, relevant organic conditions, or visual/auditory disability that could impact neuropsychological test or fMRI task performance; (2) more than 4 years of education; (3) absence of pharmacological treatment with antipsychotics, antidepressants, and/or other medications that could interfere with the fMRI examination; and (4) absence of contraindications to MRI scanning.

Before participating in the study, all participants underwent a brief clinical interview to ensure they met the inclusion/exclusion criteria. During this interview, their medical history was assessed, including previous and current health conditions, surgical procedures, and any pharmacological treatments.

In total, 59 participants were eligible for the study (32 HCs and 27 pwMS). Table 1 provides details of their demographics.

**Table 1:** Demographic and clinical characteristics of the enrolled participants.

	HCs (*n* = 32)	pwMS (*n* = 27)	*p*
Sex (F) n, %	15, 47%	18, 66%	0.161
Age (M ± SD, 95% CI)	53.90 ± 10.33, 50.11–57.63	52.74 ± 8.36, 49.43–56.05	0.643
Education, y (M ± SD, 95% CI)	14.55 ± 3.98, 13.09–16.00	13.22 ± 3.14, 11.98–14.46	0.169
EDSS	–	3.74 ± 1.72, 3.06–4.42	–
Disease duration, y	–	14.70 ± 9.10, 11.10–18.30	–

### Measures

Participants attended an individual session lasting approximately 1 h and 40 min, which included a neuropsychological evaluation battery and an MRI examination. The MRI examination included structural sequences to assess brain morphometry and rule out gross brain abnormalities, as well as the administration of the ESCoT fMRI task to examine the brain correlates of SC.

### MRI data acquisition session

The MRI protocol acquired on a 3 T Siemens Prisma scanner equipped with a 64-channel head/neck coil, included: a T1-3D magnetization prepared rapid acquisition with gradient-echo (MPRAGE) sequence with a repetition time (TR) = 2,300 ms, echo time (TE) = 3.1 ms, isotropic resolution = 0.8 mm^3^, 224 slices, a sagittal fluid-attenuated inversion recovery (FLAIR) sequence was also acquired TR/TE = 5,000/394 ms, resolution = 0.8 × 0.8 × 1 mm^3^, 176 slices; an accelerated GE sequence with TR/TE = 2,000/30 ms, resolution 3 × 3 × 3 mm^3^, multi-slice acceleration factor = 2, 52 slices, split into 2 runs composed of 247 measurements each.

The ESCoT visual stimuli were delivered using E-Prime 3.0^1^ by means of the NordicNeuroLab system consisting of an “in-room viewing device” comprising an MR-compatible display, located at the end of the gantry, and a mirror placed on the head coil allowing participants to see the monitor. Stimulus administration was synchronized with the MR acquisition by means of a dedicated device (SyncBox). The participants were trained before entering the MRI scanner by performing a short version of the fMRI experiment involving an ESCoT test video.

### fMRI task

The ESCoT fMRI task has been recently adopted and validated within a cohort of healthy participants (Isernia et al., 2024; Isernia et al., 2022). The task consists of a block design (A-B) alternating between an experimental condition (ToM reasoning) and a control condition (physical inference, PI), both of which include the same stimuli (cartoon-like video clips) presented in a randomized order. This approach allows for the exploration of neural activation related to implicit reasoning, such as reasoning elicited by the stimulus itself without any specific questions, as well as explicit reasoning, which involves reasoning prompted by questions regarding the characters’ mental states or the physical elements in the scenario (see Figure 1). The task was modified and shortened from the previous implementation, which was depicted in detail in Isernia et al. (2024), by removing open-ended questions. In fact, as shown in our previous studies validating the fMRI adaptation of the ESCoT test, open-ended questions were not effective in capturing ToM-related neural activation (Isernia et al., 2024). Furthermore, to adapt the task for administration to clinical populations with neuromotor disabilities such as pwMS, additional time (5.5 s instead of 3.5 s) was allowed for answering double-choice questions with respect to the previous version of the task.

**Figure 1:**
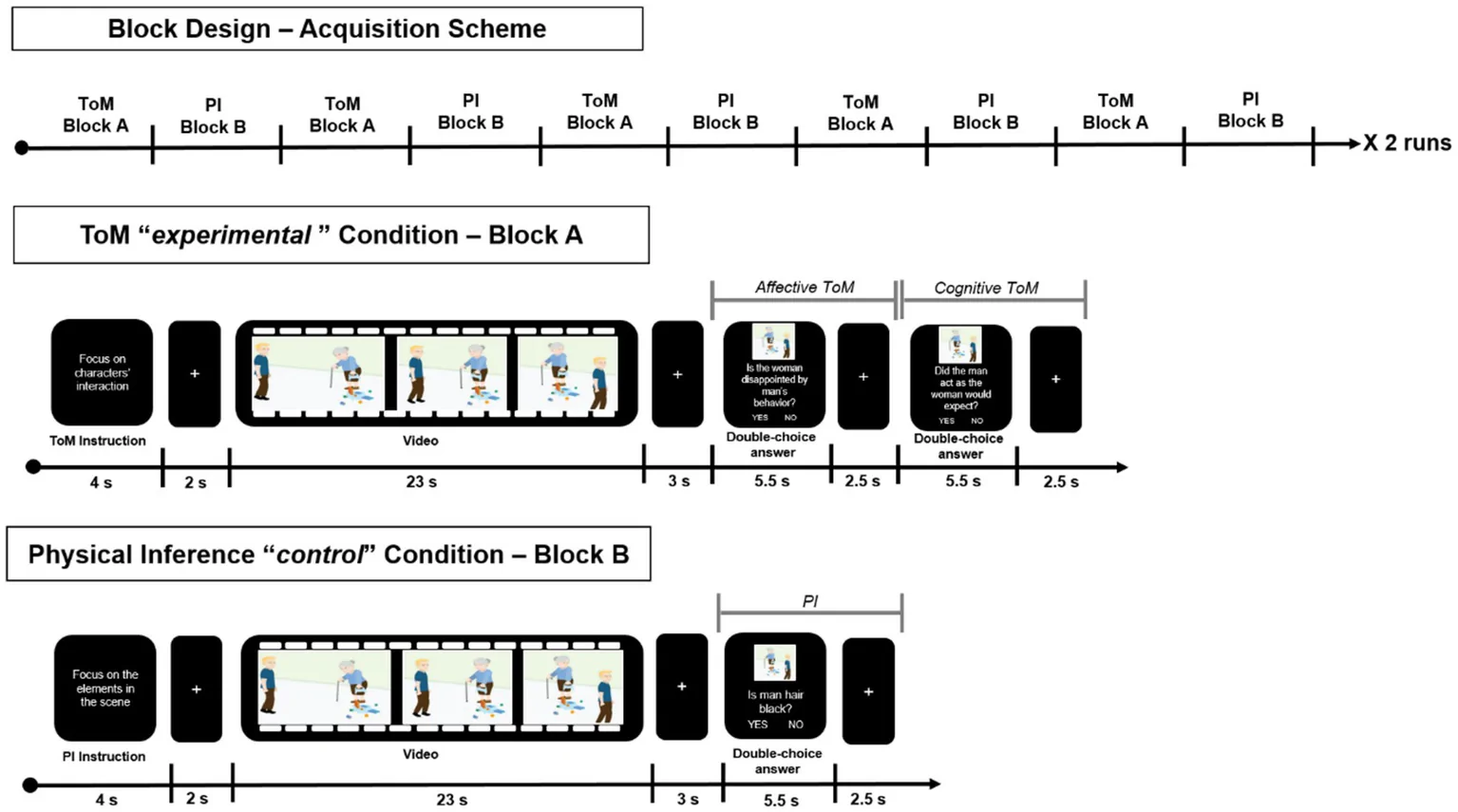
Overview of the Task-fMRI paradigm. The picture summarizes the experimental design describing items of the two blocks (namely, Block A—Theory of Mind *Experimental* Condition, and Block B—Physical Inference *Control* Condition) together with the stimulus duration, according to the order of presentation (adapted from Isernia et al., 2024).

A detailed depiction of the new version of the task is provided below and in Figure 1.

#### ToM experimental block items

1. Instructions: written instructions for the movie scene (“Focus on character interactions”), displayed on the screen for 4 s;
2. Movie: cartoon-style animation movie watching, displayed for 23 s;
3. Closed-ended affective ToM answer: a double-choice response to an affective ToM question (“Is the woman disappointed by man’s behavior?”), displayed on the screen for 5.5 s;
4. Closed-ended cognitive ToM answer: a double-choice answer to a cognitive ToM question (“Did the man act as the woman would expect?”), displayed on the screen for 5.5 s.

#### PI control block included items

1. Instructions: written instructions for the movie scene (“Focus on the elements in the scene”), displayed on the screen for 4 s;
2. Movie: cartoon-style animation movie, displayed for 23 s;
3. Closed-ended answer: a double-choice response to a PI question (“Is man’s hair black?”), displayed on the screen for 5.5 s.

All the items presented in each block were interleaved with a white fixation cross on a black background.

The task was divided into two separate but sequential runs. Each run lasted approximately 8 min, for a total fMRI task duration of 16 min. Responses to the closed-ended two-choice questions were recorded using a dedicated device, the Evoke Response Pad System (Resonance Technology Inc.). Participants indicated affirmative or negative answers by pressing a button with their index or middle finger, respectively. A threshold of 50% correct responses in the physical (control) condition was considered to ensure that participants were sufficiently attentive to the task.

### Neuropsychological sessions

The neuropsychological evaluation comprises the following:

1. Montreal Cognitive Assessment [MoCA (Conti et al., 2015)]: assessed global cognitive level; the total score, ranging from 0 to 30, was adjusted for age and education according to Santangelo et al. (2015).
2. Trail Making Test [TMT (Giovagnoli et al., 1996)]: evaluated shifting frontal executive functions; scores were based on performance times from both TMT parts A and B and were adjusted for age and education according to Giovagnoli et al. (1996).
3. Stroop Color-Word Test (Strauss et al., 2006): assessed inhibitory frontal executive functions; both performance time and error scores were adjusted for age and education according to Caffarra et al. (2002).
4. Symbol Digit Modality Test [SDMT (Sheridan et al., 2006)]: evaluated processing speed and attention; the score was adjusted according to Rao (1990).
5. Digit Span Forward and Backward (Monaco et al., 2013) test: assessed short-term and working memory, respectively; total scores were adjusted for sex, age, and education according to Monaco et al. (2013).
6. Yoni-48 task (Isernia et al., 2022): evaluated cognitive and affective, first-order and second-order ToM; accuracy and response time scores were adjusted for sex, age, and education according to Isernia et al. (2022).
7. Reading the Mind in the Eyes test [ET (Maddaluno et al., 2022)]: evaluated ToM using photographs of eyes expressing complex mental states; the score (ranging from 0 to 36) was adjusted according to Maddaluno et al. (2022).

### Statistical analysis

#### Sample size estimation

According to the guidelines of Szucs and Ioannidis (2020), we estimated the sample size for the present study by considering both previous evidence in the literature and performing an *a priori* calculation using R software (version 4.4.1) with the wp.kanova package. Based on our previous publication (Isernia et al., 2024) on the application and validation of the fMRI task, a sample size of 35 participants was sufficient to detect ToM neural activations during the task. Moreover, the *a priori* calculation suggested a sample size of 50 participants for performing a full factorial model analysis (two-level between-subjects factor: group; two-level within-subjects factor: condition; group*condition interaction), assuming an alpha threshold of 0.05, a 1-beta power of 0.80, and a medium effect size f of 0.4.

#### MRI data processing

The FLAIR images were visually inspected by an experienced neuroradiologist to exclude gross brain abnormalities and, using a semiautomatic tool, JIM,^2^ to segment WM hyperintensities. The lesion masks derived from the segmentation were then used to compute the lesion load (for pwMS only) and to perform lesion filling of the MPRAGE images.

The MPRAGE anatomical volumes were then preprocessed using the FMRIB Software Library v6.0 (FSL, https://fsl.fmrib.ox.ac.uk/fsl/fslwiki/) as follows: bias field correction, brain extraction, and lesion filling. The individual volumes were subsequently used as anatomical references for the registration of functional volumes as described below.

Functional MRI data were preprocessed using the Statistical Parametric Mapping toolbox (SPM12, https://www.fil.ion.ucl.ac.uk/spm/) running on MATLAB (MathWorks, https://it.mathworks.com/products/matlab.html), following a standard pipeline. Specifically, after discarding the first 10 acquired volumes to account for magnetization stabilization, the two runs were preprocessed together as different sessions following these steps: motion correction and realignment of functional volumes to an average reference volume; co-registration with the individual anatomical volumes (MPRAGE); tissue segmentation and normalization to the standard MNI template; and smoothing (8 mm full width at half-maximum isotropic Gaussian). The preprocessed functional volumes served as input to the following first-level statistical analyses.

#### fMRI statistics

The performance during the fMRI task was recorded, and between-group differences were investigated by comparing accuracy and reaction times using Mann–Whitney U and independent-samples t-tests, respectively.

#### First-level analyses

The first-level analysis was conducted using the general linear model (GLM) to fit the statistical model on the BOLD response. Every item belonging to either the ToM experimental condition or PI control condition was modeled as a single event, specifically movie viewing (following ToM or PI instructions) and double-choice answering (AToM, CToM, or PI questions), which were included as regressors of interest. The six motion parameters were also included in the model as nuisance regressors. Four different contrasts were considered to compare items between the two conditions (i.e., ToM Experimental condition and PI Control condition). At the individual level, main effect statistical maps were derived for all the modeled events, namely for the following conditions: movie watching following ToM instructions (implicit reasoning), movie watching following PI instructions, ToM double choice answering (explicit reasoning), and PI double choice answering.

#### Second-level analyses

Second-level analyses were designed according to a full-factorial model comprising two factors of two levels each, namely factor 1: (HC group vs. pwMS group) and factor 2: (experimental ToM condition vs. Control PI condition), which were compared separately for implicit (movie watching) and explicit (double choice answering) reasoning. For each comparison, contrasts evaluating the overall effect of either group (HC or pwMS) or conditions (ToM or PI) were computed. Results were restricted to the GM region, using a WM-exclusive mask derived from SPM standard tissue probability maps, thereby limiting the analysis to regions where task-related BOLD responses are physiologically expected. Statistically significant clusters were selected considering a threshold equal to p_FWE_ < 0.05 and a minimum cluster size equal to 50 voxels. Since this analysis already implemented FWE control, the results of the following correlations were not further corrected for multiple comparisons. Specifically, each of the above-mentioned neural activation clusters was correlated with neuropsychological test scores in each group separately (Spearman’s ρ), in order to examine the association between ToM-related neural efficiency, ToM performance, and executive function level (R software, retrieved from https://www.r-project.org/, version 4.3.3). Finally, a simultaneous multiple regression model (fMRI ToM task accuracy ~ sex + age + group + SDMT + TMTB + activation cluster) was conducted to test ToM-related compensatory activation mechanisms by predicting fMRI task ToM performance from sex, age, group (PwMS; HCs), executive function performance (SDMT and TMTB scores), and cluster neural activation.

## Results

### Participants

Among the eligible participants for the research, three pwMS were excluded from analyses due to a low (≤ 50%) accuracy in the control (physical inference) condition of the fMRI task.

In total, 56 participants (32 HCs and 24 pwMS) constituted the study sample. Table 2 reports the sample characteristics and indicates that the two groups were equal in terms of demographics. However, they differed in executive function, divided attention, and processing speed (pwMS < HCs) and showed a trend toward underperformance by pwMS in affective and second-order ToM (see Table 3).

**Table 2:** Demographic and clinical characteristics of the participants included in the analyses.

	HCs (*n* = 32)	pwMS (*n* = 24)	*p*
Sex (F) n, %	16, 50%	15, 63%	
Age (M ± SD, 95% CI)	54.25 ± 10.35, 50.52–57.98	52.33 ± 7.99, 48.96–55.71	0.454
Education, y (M ± SD, 95% CI)	14.66 ± 3.96, 13.23–16.09	13.38 ± 3.27, 11.99–14.75	0.203
EDSS	–	3.79 ± 1.82, 3.02–4.56	–
Disease duration, y	–	15.38 ± 9.38, 11.41–19.34	–
Disease-modifying therapy, n (%)			
Yes		14 (58)	
No		10 (42)	
Immunomodulatory			
Interferon beta, n (%)		1 (4)	
Glatiramer acetate, n (%)		4 (17)	
Fingolimod, n (%)		2 (8)	
Ozanimod, n (%)		2 (8)	
mAb			
Ocrelizumab, n (%)		1 (4)	
Natalizumab, n (%)		1 (4)	
Teriflunomide, n (%)		2 (8)	
Dimethyl fumarate, n (%)		1 (4)	
Lesion load [mm^3^] (M ± SD, 95% CI)	-	11,798 ± 12,895, 6,639–16,957	-^a^

HCs, healthy controls; pwMS, people with multiple sclerosis; M, mean; SD, standard deviation; 95% CI, 95% confidence interval; ToM, theory of mind.

a Lesion load was not assessed in the HC cohort.

**Table 3:** Neuropsychological characteristics of the participants.

	HC (*n* = 32)	pwMS (*n* = 24)	Cut-off	*p*
MoCA (M ± SD, 95% CI)	23.32 ± 2.21, 22.51–24.12	23.20 ± 1.95, 22.38–24.02	<17.37	0.841
TMTA (M ± SD, 95% CI)	22.95 ± 9.27, 19.49–26.42	29.28 ± 11.52, 29.28–34.15	≥94	0.030
TMTB (M ± SD, 95% CI)	61.19 ± 33.15, 73.57–48.81	80.39 ± 43.86, 61.87–98.91	≥283	0.073
Stroop time (M ± SD, 95% CI)	17.03 ± 6.04, 14.82–19.25	22.21 ± 12.25, 17.04–27.39	>36.92	0.045
Stroop errors (M ± SD, 95% CI)	0.17 ± 0.69, 0.00–0.42	0.19 ± 0.55, 0.00–0.42	<4.24	0.917
Digit span F (M ± SD, 95% CI)	6.06 ± 1.01, 5.69–6.43	5.78 ± 1.12, 5.30–6.25	<4.25	0.331
Digit span B (M ± SD, 95% CI)	4.67 ± 1.35, 4.17–5.16	4.41 ± 1.26, 3.88–4.94	<2.64	0.476
SDMT (M ± SD, 95% CI)	53.82 ± 9.93, 50.17–57.46	45.68 ± 9.54, 41.65–49.71	<34.20	0.003
Eyes test (M ± SD, 95% CI)	26.07 ± 2.96, 24.98–27.15	25.21 ± 3.31, 23.77–26.64	≤15.72	0.319
Yoni-48 task (M ± SD, 95% CI)				
ACC	0.87 ± 0.13, 0.82–0.92	0.81 ± 0.13, 0.76–0.87	0.60	0.107
Affective ToM	18.06 ± 3.04, 16.94–19.17	16.89 ± 2.50, 15.87–18.03	–	0.086
Cognitive ToM	18.49 ± 3.12, 17.35–19.63	17.25 ± 3.57, 15.71–18.80	–	0.157
First-order ToM	15.91 ± 0.34, 15.79–16.03	15.37 ± 2.06, 14.48–16.27	–	0.213
Second-order ToM	20.99 ± 4.95, 19.17–22.80	18.79 ± 4.99, 16.64–20.95	–	0.099

HCs, healthy controls; pwMS, people with multiple sclerosis; ACC, Yoni-48 Accuracy score; Digit Span B, digit span backward; Digit Span F, digit span forward; EDSS, Expanded Disability Status Scale; MoCA, Montreal Cognitive Assessment; M, mean; SD, standard deviation; SDMT, symbol digit modalities test; TMT, trail making test; 95% CI, 95% confidence interval; ToM, theory of mind.

### fMRI task results

The results of performance during the fMRI task revealed a significant between-group difference in terms of both accuracy (*p* = 0.021) and reaction times (*p* = 0.04). Specifically, pwMS were slower and more error-prone than HCs.

Figure 2 shows the localization of the GM significant neural activation (p_FWE_ < 0.05, k = 50 voxels), detailed in Table 4, obtained through factorial modeling of ToM answering, independently of the groups. The ESCOT task resulted in effective activation of ToM-related circuits in the whole sample. However, no group effect emerged from the analyses.

**Figure 2:**
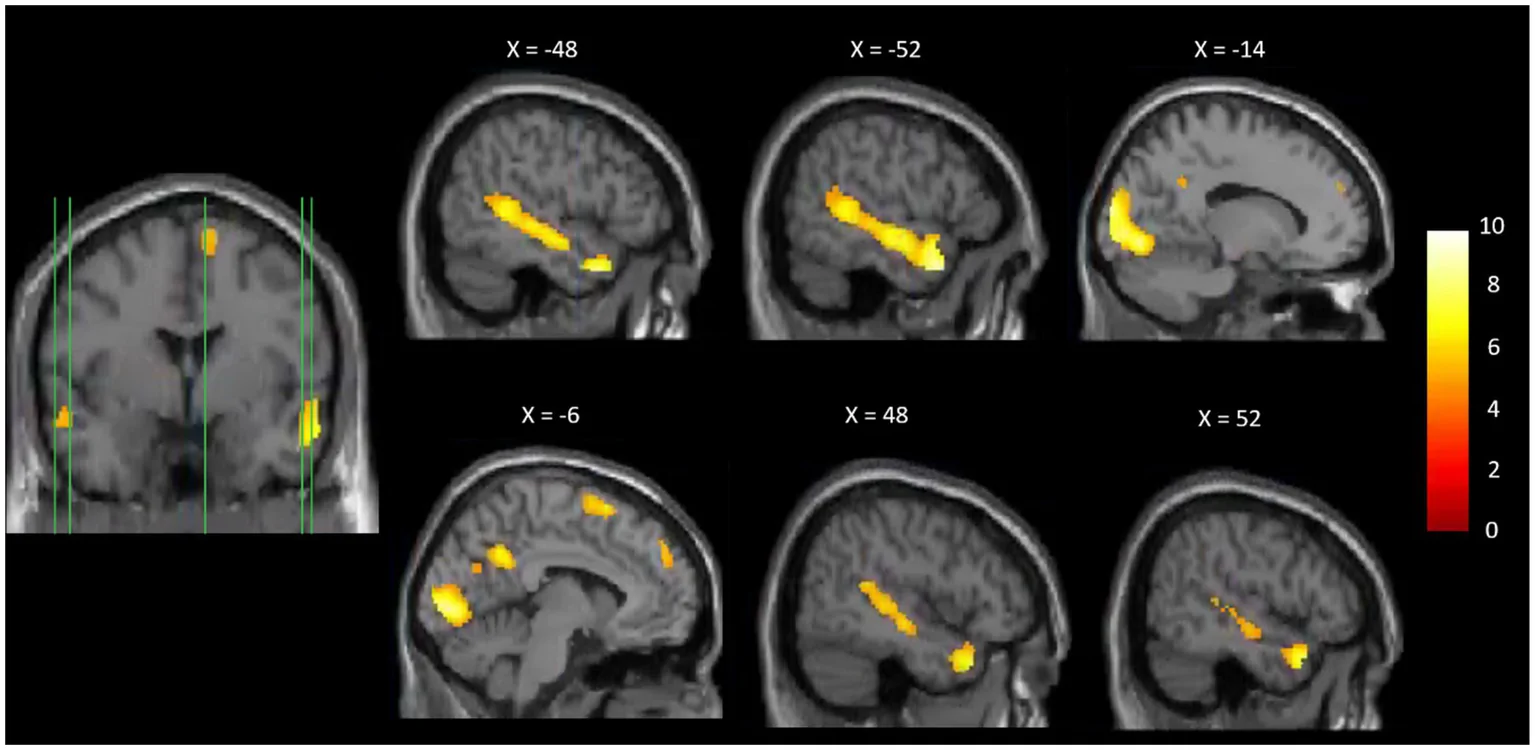
Significant neural activation resulting from the full-factorial analysis comparing explicit ToM reasoning with PI reasoning in both groups. Results are color-coded according to *t*-values, as indicated by the color bar to the right.

**Table 4:** Mapping of significant results derived from the second-level full factorial analyses comparing the ToM closed-question answering condition (experimental condition) with the physical inference condition (control condition).

p(FWE-corr)	equivk	T	x, y, z [mm]	AAL/BA Label
0.002	55	10	−18, −94, 16	L Middle Occipital C
				Middle occipital G, Cuneus, BA 18, 19
<0.001	1,151	9.11	−50, 12, 28	L Middle Temporal Pole
				Superior temporal G, middle temporal G, superior and middle temporal pole, BA 21, 22, 38
<0.001	435	7.86	−14, −76, −10	L Lingual G
				Calcarine C, Cuneus, BA 18
<0.001	169	7.84	22, –92, 18	R Sup Occipital C
				Cuneus, middle occipital G, BA 18, 19
<0.001	521	7.61	52, 16, −26	R Middle Temporal Pole
				Superior temporal G, middle temporal G, BA 21, 38
<0.001	537	7.23	−8, −52, 34	L Precuneus
				Cingulate G, BA 7, 31
<0.001	152	6.49	−6, 14, 64	L Supplementary Motor Area
				Superior frontal G, BA 6
<0.001	103	5.97	−8, 52, 32	L Superior Medial Frontal C
				Superior frontal G

The results have been localized according to both Automated Anatomical Labeling (AAL) and Brodmann (BA) Atlases using the xjView toolbox (https://www.alivelearn.net/xjview), part of SPM12. C, cortex; G, gyrus; BA, Brodmann Area Atlas; AAL, Automated Anatomical Labeling Atlas; L, left; R, right.

### Correlations of fMRI brain activation with ToM neuropsychological measures

Bivariate correlations were conducted to examine the relationship between neural activation patterns during the ToM condition of the fMRI task (specifically across eight neural clusters: left middle occipital cortex, right superior occipital cortex, left middle temporal pole, right middle temporal pole, left supplementary motor area, left precuneus, left superior medial frontal area, and left lingual region) and ToM performance on neuropsychological measures (ET and Yoni task) in both the pwMS and HC groups. Interestingly, a significant inverse association emerged exclusively in the pwMS group between Yoni task accuracy and activation in the left superior medial frontal area: the greater recruitment of this frontal region during the ToM fMRI task was associated with lower accuracy on the Yoni task (rho = −0.447, *p =* 0.032, see Figure 3), which may suggest a compensatory mechanism (Table 5).

**Figure 3:**
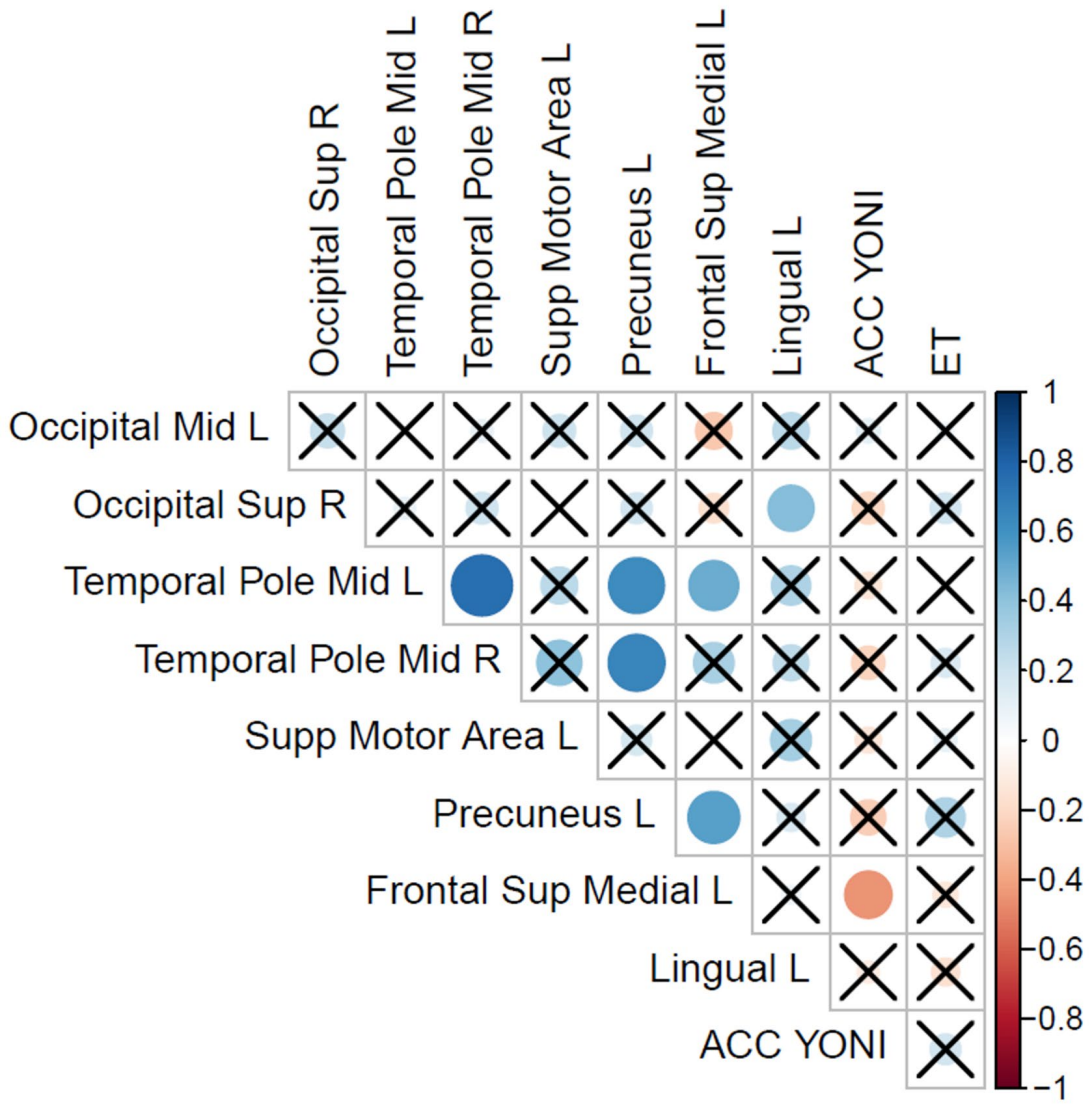
Spearman correlations between neural activation patterns during the ToM condition of the fMRI task and the ToM neuropsychological assessment measures. Circle size reflects the magnitude of the correlation coefficient; larger circles indicate stronger correlations, while circle color reflects the direction of the association between variables: Hot colors indicate a negative correlation, and cool colors reflect a positive correlation. Crossed-out circles indicate non-significant correlations (*p*-value ≥ 0.05). ACC YONI = Yoni task accuracy score; ET = eyes test score; frontal sup medial L = left superior medial frontal area; lingual L = left lingual region; occipital Mid L = left middle occipital; occipital Sup R = right superior occipital; precuneus L = left precuneus; supp motor area L = left supplementary motor area; temporal pole mid L; left middle temporal pole; temporal pole mid R = right middle temporal pole.

**Table 5:** Multiple regression testing the ToM-related compensatory mechanism.

	Estimate	SE	t	Targeted *p*	F	omnibus *p*
Intercept	1.07	0.14	7.86	<0.001	4.66	<0.001
SDMT	0.00	0.00	0.08	0.935		
TMT-B	0.00	0.00	−3.11	0.003		
dmPFC	−0.04	0.02	−2.49	0.016		
Age	−0.00	0.00	−2.30	0.026		
Sex	0.05	0.02	2.00	0.052		
Group	−0.04	0.02	−1.61	0.115		

dmPFC, dorsomedial prefrontal cortex neural activation cluster; SDMT, Symbol Digit Modality Test; SE, standard error; TMT-B, Trail Making Test-part B.

To further investigate this mechanism, a simultaneous multiple regression model was conducted to predict fMRI ToM task accuracy from mean activation within the dmPFC cluster, executive function level (TMT-B and SDMT scores), group (pwMS vs. HCs), sex, and age. The overall model was robust and statistically significant (R^2^ = 0.373; *F* = 4.66; omnibus *p* < 0.001). Among the predictors, executive function level and dmPFC neural activation significantly explained variance in fMRI ToM task accuracy: greater dmPFC activation and better TMT-B performance were associated with higher ToM accuracy, with targeted *p*-values of 0.016 and 0.003, respectively.

## Discussion

This is the first study to investigate the neural underpinnings of SC in pwMS and to demonstrate the validity of the ESCoT as an effective fMRI task paradigm for measuring ToM deficits in a clinical population. Our working hypothesis posited that pwMS would exhibit aberrant neural activation patterns during the SC task compared with neurotypical individuals and would rely on compensatory mechanisms to maintain SC performance.

Contrary to our expectations, the main findings did not reveal significant differences in neural activation between pwMS and HCs during the SC task. Both groups effectively recruited core regions of the social brain associated with ToM reasoning in the ESCoT fMRI paradigm (Isernia et al., 2024), including the left precuneus, superior medial frontal region, and bilateral temporal poles.

This outcome may be partly attributed to the mild disability level of our pwMS participants (mean EDSS score = 3.74). Notably, their performance on neuropsychological ToM measures (Yoni task and ET) was within the normal range, with only slight underperformance observed in affective and second-order ToM scores on the Yoni task. Overall, these findings suggest that when SC difficulties in pwMS are still subtle, neural efficiency remains largely intact. Consistent with this interpretation, although pwMS showed significantly lower accuracy and longer reaction times during the fMRI task, these behavioral differences likely reflect difficulties in ancillary ToM-related cognitive functions, such as processing speed and attention. Importantly, these behavioral impairments were not accompanied by significant between-group differences in task-related neural activation. This pattern is further supported by the neuropsychological profile of pwMS, which is characterized by genuine deficits only in non-ToM functions, and only a trend toward lower performance than healthy individuals on ToM tasks, such as the Yoni task. This apparent dissociation suggests that subtle behavioral changes may occur despite a preserved overall recruitment of the mentalizing network. Interestingly, when examining the relationship between ToM-related neural activation during the fMRI task and behavioral performance on neuropsychological measures independently evaluated, we identified a potential compensatory mechanism in pwMS. Specifically, increased recruitment of the left dorsomedial prefrontal cortex (dmPFC) during the ToM condition was inversely associated with performance on the Yoni task. Although we did not find a significant group effect, this inverse correlation suggests that greater neural activation in this frontal area was linked to lower behavioral accuracy on the ToM test, possibly reflecting an early compensatory mechanism aimed at counteracting emerging socio-cognitive difficulties. The role of the frontal regions as auxiliary hubs for higher-order socio-cognitive functions has been highlighted in established ToM network models (Abu-Akel and Shamay-Tsoory, 2011). Specifically, the dmPFC belongs to the “execution nodes” of the cognitive ToM network and has been proposed to support higher-order inferential computations required for integrating and manipulating mental state representations (Abu-Akel and Shamay-Tsoory, 2011). Previous research has also shown that the disrupted connectivity between this region and other social brain areas can lead to pronounced ToM deficits (Isernia et al., 2022). Thus, in the early phases of MS-related cognitive impairment, enhanced recruitment of the left superior frontal cortex may serve as a compensatory response to subtle SC underperformance. This interpretation is further supported by the fact that the inverse relationship was exclusively observed within the pwMS group and not in HCs, suggesting that dmPFC recruitment reflects disease-related inter-individual differences. Notably, the increased activation of the dmPFC during ToM tasks in pwMS may reflect the engagement of executive function hubs to support SC performance. Particularly, prefrontal areas are widely recognized as a core neural substrate for executive functions (Jobson et al., 2021; Friedman and Robbins, 2022), and their increased activation has been documented in individuals with cognitive impairments (Rocca et al., 2014). Accordingly, pwMS who exhibit early impairment in executive functioning and rely more heavily on executive neural circuits, such as the dmPFC, tend to show greater involvement of this area during SC tasks. These findings underscore the interplay between two neurocognitive domains, SC and executive function, suggesting a crosstalk that may prompt compensatory strategies. Although the present cross-sectional design does not allow us to directly demonstrate compensatory mechanisms, the observed inverse association is compatible with the concept of neural efficiency and with adaptive functional recruitment reported in pwMS (Filippi and Rocca, 2009).

In addition, the dmPFC is considered a core ‘computational’ hub of the mentalizing networks, which is engaged when individuals integrate, manipulate, and evaluate others’ mental states, particularly under conditions requiring explicit perspective taking and complex social reasoning rather than simple mental state attribution, such as in ESCoT scenarios (Abu-Akel and Shamay-Tsoory, 2011; Amodio and Frith, 2006; Van Overwalle, 2011). Thus, the neural activity within this region likely reflects the computational effort associated with mental-state reasoning rather than the successful execution of the task itself. In this framework, the increased recruitment of this area to sustain higher ToM performance could be linked to the increased computational effort required for more complex reasoning in pwMS (Filippi and Rocca, 2009). This notion is further supported by the results of the multiple regression model, which tested the role of dmPFC efficiency and executive function in compensating for ToM performance during the fMRI task. Specifically, increased activation in this neural cluster, together with poorer performance on the executive function test (TMT-Part B), was associated with lower fMRI task performance. This compensatory mechanism had already been proposed in previous research (Isernia et al., 2020; Isernia et al., 2022), suggesting the involvement of frontal executive loops in ToM deficits in pwMS. Interestingly, the regression model identified a significant contribution of set shifting, but not processing speed, in explaining ToM accuracy during the fMRI task. This finding is in line with recent evidence suggesting that executive functions, and cognitive flexibility in particular, are more closely related to SC than processing speed. While processing speed may facilitate the efficient processing of social information, the ability to shift between perspectives, update mental representations, and flexibly adapt to changing social contexts appears to constitute a more central mechanism underlying SC. This interpretation is consistent with a recent systematic review demonstrating that cognitive flexibility is among the executive processes most strongly associated with ToM and other domains of SC across development (Badarneh et al., 2024).

From a clinical perspective, the association between greater dmPFC recruitment and preserved ToM performance underscores the potential value of functional neuroimaging for detecting subtle social cognitive changes and compensatory mechanisms at an early stage of the disease, potentially preceding overt behavioral impairment. Such functional activation profiles may also contribute to the early characterization of cognitive impairment, support the development of targeted rehabilitation strategies to strengthen neural hubs involved in SC, and provide a neurobiological basis for evaluating whether interventions promote adaptive rather than maladaptive neuroplastic changes.

Finally, the absence of between-group differences with respect to the HC cohort could be explained by the high degree of inter-individual variability in neural recruitment observed in the MS cohort, as previously reported in the literature (Pfaff et al., 2019; Pirastru et al., 2026) and characterizing the emotional and social cognitive domain itself (Isernia et al., 2024; Pirastru et al., 2023).

This study is not without limitations. First, the sample size is relatively small, and larger cohorts will be needed to ensure higher statistical power and validate these findings. Second, we focused exclusively on ToM as a facet of SC, potentially overlooking other domains where aberrant neural mechanisms may emerge earlier in the disease course. Furthermore, although our participants did not exhibit overt SC deficits on neuropsychological tests, prior literature indicated that SC impairment can occur even in early stages of MS. Further research is warranted to explore neural mechanisms across diverse SC phenotypes in MS. Finally, our participants presented with mild disability, which allowed us to investigate ToM-related neural efficiency in the early stages of MS, and these findings cannot be generalized to individuals with more severe disease. Future studies may adopt longitudinal designs or different experimental settings (i.e., fMRI acquired in a resting condition) to include participants across different disability stages to better characterize how ToM functioning evolves over the course of the disease.

In conclusion, our findings suggest that in individuals with MS with low disability levels, the neural efficiency of the SC network is preserved, supporting performance comparable to that of HCs. However, early compensatory mechanisms may already be in place, particularly involving increased recruitment of frontal regions during ToM reasoning. Specifically, our findings suggest that dmPFC activation may not simply reflect successful ToM processing, but rather the computational demand required by mentalizing reasoning. This interpretation aligns with neural efficiency models and compensatory functional reorganization mechanisms, within the context of MS, where inter-individual variability is a hallmark of disease-related brain plasticity. Most importantly, the lack of significant between-group differences suggests that the compensatory role of the dmPFC may represent a more sensitive marker of social cognitive functioning than average group activation differences. Finally, the study also provides preliminary support for the use of the ESCoT paradigm as an ecologically valid assessment approach capable of detecting early ToM deficits in the clinical population and supporting the development of targeted rehabilitation strategies promoting adaptive neuroplastic changes.

Future studies should investigate these mechanisms across varying cognitive profiles and stages of disease progression.

## Data Availability

The raw data supporting the conclusions of this article will be made available by the authors, upon request to the corresponding author.

## Footnotes

1
https://pstnet.com/products/e-prime/

2
https://www.xinapse.com/
