# Supplementary material for: Left dorsomedial prefrontal cortex hyperactivation reflects early compensation for emerging social cognition difficulties in multiple sclerosis
Source: Front Psychol. 2026 Sep 29;17:1905506. doi: 10.3389/fpsyg.2026.1905506 (PMC13657971; doi:10.3389/fpsyg.2026.1905506)
Supplement: Supplementary file 1 [file Table_1.DOCX]

**Task reproducibility**

METHODS: Finally, to assess within-session reproducibility, the two functional runs acquired from each participant belonging to the HC group were treated as independent acquisitions and preprocessed separately. At the first level, contrast maps for implicit and explicit ToM processing (i.e., movie watching and ToM closed-ended question answering, respectively) were estimated independently for each run. Paired t-tests were then performed to compare the corresponding contrast estimates across runs. Statistical significance was assessed at a family-wise error (FWE)-corrected threshold of p_FWE_ < 0.05.

RESULTS: No significant differences were observed for either contrast (p_FWE_ < 0.05), suggesting good within-session reproducibility of the task.
